# Dissection of Signaling Events Downstream of the c-Mpl Receptor in Murine Hematopoietic Stem Cells Via Motif-Engineered Chimeric Receptors

**DOI:** 10.1007/s12015-017-9768-7

**Published:** 2017-09-25

**Authors:** Koichiro Saka, Chen-Yi Lai, Masanori Nojima, Masahiro Kawahara, Makoto Otsu, Hiromitsu Nakauchi, Teruyuki Nagamune

**Affiliations:** 1 Department of Chemistry and Biotechnology, Graduate School of Engineering, University of Tokyo, 7-3-1 Hongo, Bunkyo-ku, Tokyo, 113-8656 Japan; 2 Division of Stem Cell Therapy, Center for Stem Cell Biology and Regenerative Medicine, Institute of Medical Science, University of Tokyo, 4-6-1 Shirokanedai, Minato-ku, Tokyo, 108-8639 Japan; 3 Division of Stem Cell Processing / Stem Cell Bank, Center for Stem Cell Biology and Regenerative Medicine, Institute of Medical Science, University of Tokyo, 4-6-1 Shirokanedai, Minato-ku, Tokyo, 108-8639 Japan; 4 Division of Advanced Medicine Promotion, Advanced Clinical Research Center, Institute of Medical Science, University of Tokyo, 4-6-1 Shirokanedai, Minato-ku, Tokyo, 108-8639 Japan; 5 Institute for Stem Cell Biology and Regenerative Medicine, Stanford University School of Medicine, Stanford, CA 94305 USA

**Keywords:** Hematopoietic stem cells, C-Mpl, Chimeric receptors, Signal transduction

## Abstract

Hematopoietic stem cells (HSCs) are a valuable resource in transplantation medicine. Cytokines are often used to culture HSCs aiming at better clinical outcomes through enhancement of HSC reconstitution capability. Roles for each signal molecule downstream of receptors in HSCs, however, remain puzzling due to complexity of the cytokine-signaling network. Engineered receptors that are non-responsive to endogenous cytokines represent an attractive tool for dissection of signaling events. We here tested a previously developed chimeric receptor (CR) system in primary murine HSCs, target cells that are indispensable for analysis of stem cell activity. Each CR contains tyrosine motifs that enable selective activation of signal molecules located downstream of the c-Mpl receptor upon stimulation by an artificial ligand. Signaling through a control CR with a wild-type c-Mpl cytoplasmic tail sufficed to enhance HSC proliferation and colony formation in cooperation with stem cell factor (SCF). Among a series of CRs, only one compatible with selective Stat5 activation showed similar positive effects. The HSCs maintained ex vivo in these environments retained long-term reconstitution ability following transplantation. This ability was also demonstrated in secondary recipients, indicating effective transmission of stem cell-supportive signals into HSCs via these artificial CRs during culture. Selective activation of Stat5 through CR ex vivo favored preservation of lymphoid potential in long-term reconstituting HSCs, but not of myeloid potential, exemplifying possible dissection of signals downstream of c-Mpl. These CR systems therefore offer a useful tool to scrutinize complex signaling pathways in HSCs.

**Electronic supplementary material:**

The online version of this article (10.1007/s12015-017-9768-7) contains supplementary material, which is available to authorized users.

## Introduction

Hematopoietic stem cells (HSCs) are characterized by the ability to self-renew and to differentiate into blood cells of multiple lineages [1–4]. Transplantation of HSCs permits treatment of various disorders, including hematologic malignancies and primary immune deficiency diseases [5, 6]. Much effort has been made to achieve ex vivo HSC expansion [7–9], or to enhance HSC abilities [10], aiming at improvement of transplantation outcomes. Almost all expansion protocols so far use multiple cytokines, generally including a combination of stem cell factor (SCF) and thrombopoietin (TPO). These two cytokines in combination induce *in vitro* self-renewal in purified murine HSCs [11]. To understand how signals downstream from these cytokine receptors affect stem cell activity remains critical to better clinical use of HSCs. The receptors of SCF and TPO are cKit and c-Mpl, respectively [12, 13]. With c-Mpl, ligand binding results sequentially in receptor oligomerization, activation of Janus kinase (JAK), phosphorylation of tyrosine residues in the receptor intracellular domain, and activation of downstream signaling molecules [14, 15]. Of note is that the amino acid sequence surrounding the receptor tyrosine residue determines specificity for binding of signaling molecules [16]; *e.g*., STAT5 binds to the consensus motif YXXL [17]. Using the known consensus motifs in cytokine receptors, we established a chimeric receptor (CR) system with single-chain Fv (ScFv)/cytokine receptor chimeras capable of motif-specific recruitment of downstream molecules upon stimulation with the artificial ligand BSA-Fluo, *viz*., fluorescein (Fluo)-conjugated bovine serum albumin (BSA) [18–20]. With the use of a prototype CR, ScFv/c-Mpl (S-Mpl), we showed that BSA-Fluo stimulation in Ba/F3 cells activated the signaling molecules Stat1, Stat3, Stat5, PI3K, and Shc, all known as TPO-related signal proteins [20, 21]. In contrast, we observed distinct patterns of signal activation in cells expressing one of several CRs derived from S-Mpl that incorporated unique tyrosine motifs. For example, stimulation of the S-Mpl CR containing a motif known to recruit STAT5 led to highly specific phosphorylation of Stat5 [20].

Several different approaches so far have been taken for the analysis of cytokine signaling in hematopoietic cells [13]. Elegant studies attempted dissection of signals downstream of c-Mpl with genetically engineered receptors, mostly using hematopoietic cell lines [22, 23]. These approaches resemble ours in some respects. Importance of each signal component for stem cell activity, however, can only be assessed with studies using primary HSCs in transplantation assays. For this reason, genetically modified mice have been particularly valuable. Mice are described that either lack the entire c-Mpl receptor [24–26] or express a truncated c-Mpl devoid of the distal 60 amino acid residues [27]. Studies in these mice have revealed roles of c-Mpl signaling in HSCs under both steady-state and stressed-state conditions, with meaningful signal dissection in the latter [27]. Detailed analysis, however, must await generation of wide varieties of transgenic mice that incorporate different mutant c-Mpl receptors – not totally unfeasible, but requiring much time to achieve comprehensive understanding of complex signaling pathways in HSCs [28].

In this study, we deployed recent technological advances to dissect c-Mpl signaling based on motif-engineered chimeric receptors in primary HSCs. Using our unique CR system [18–20] and retroviral-mediated transduction techniques developed in our laboratory [29, 30], we investigated how selective/specific activation of signaling molecules downstream of c-Mpl affected abilities of highly purified HSCs in short-term culture.

## Materials and Methods

### Animals

C57BL/6 (B6)-Ly5.1, B6-Ly5.2, and B6-Ly5.1/Ly5.2 mice were from Japan SLC (Shizuoka, Japan). The Animal Experiment Committee of the Institute of Medical Science, University of Tokyo, approved all animal experiments in this study.

### Purification of Mouse HSCs

CD34^−/low^c-Kit^+^Sca-1^+^Lin^−^ (CD34^−^ KSL) HSCs were purified from bone marrow (BM) of B6-Ly5.1 mice [3, 29, 31]. Stringent gating strategies yield CD34^−^KSL HSCs that are ~ 100% CD48-negative / ~70–80% CD150-positive [32], *viz*., with stem cell purity close to that achieved using SLAM family markers [1]. BM cells obtained from 8- to 12-week-old mice were stained with allophycocyanin (APC)-conjugated anti-c-Kit antibodies (BioLegend, San Diego, CA) and c-Kit^+^ cells were enriched using anti-APC magnetic beads and columns (Miltenyi Biotec, Bergisch Gladbach, Germany). These cells were then stained with a lineage-marker cocktail consisting of biotinylated anti-Gr-1, -Mac-1, -Ter119, -B220, -CD4, -CD8, and -IL7R (interleukin-7 receptor) monoclonal antibodies (mAb) (e-Bioscience, San Diego, CA), fluorescein isothiocyanate (FITC)-conjugated anti-CD34, APC-conjugated anti-c-Kit, PE-conjugated Sca-1 mAbs, and streptavidin-Alexa 780, and were subjected to cell sorting. Highly purified long-term HSCs are defined as a CD34-negative/dull fraction within KSL cells [32]. Information on mAbs is available as Supplementary Table 1.

### Cell Lines

Two retroviral packaging cell lines were used; 293GP cells [33] were cultured in Dulbecco’s modified Eagle’s medium (DMEM; Nissui Pharmaceutical, Tokyo, Japan) supplemented with 10% fetal bovine serum (FBS) and 293GPG cells [34] were cultured in DMEM supplemented with 10% FBS, 2 μg/ml puromycin (Sigma, St Louis, MO), 300 μg/ml G418 (Calbiochem, Darmstadt, Germany), and 1 μg/ml tetracycline (Sigma).

### Construction of Retroviral Vectors Encoding cDNA for Chimeric Receptors (CRs)

Detailed methods are described [20]. The pGCDNsam-IRES-EGFP (I/E) backbone vector [30] was used for construction of a series of CR vectors and also used unmodified as a control vector (Mock). Construction of the prototype CR vector pGCDNsam-S-Mpl-I/E is described (S, Single-chain Fv) [18]. The receptor produced by this construct is called S-Mpl-WT, as it has the full-length cytoplasmic domain of wild type (WT) c-Mpl (Supplementary Fig. 1a, WT). The pGCDNsam-S-Mpl_t69_-no motif vector was constructed via mutagenesis (Supplementary Fig. 1b) [18]. With this vector, the CR called S-Mpl-NM contains only the JAK binding domain of c-Mpl (c-Mpl cytoplasmic domain 1–69; Supplementary Fig. 1a, NM for no motif). A series of vector constructs was generated by inserting double-strand oligonucleotides downstream from the JAK binding domain to express S-Mpl receptors capable of specifically activating one target signal transducer due to the presence of an individual tyrosine motif in each (S-Mpl-STAT1; S-Mpl-STAT3; S-Mpl-STAT5; S-Mpl-PI3K, and S-Mpl-Shc; Supplementary Fig. 1c). As shown in Supplementary Fig. 1a, each expressed CR has a structure composed of a hemagglutinin (HA)-tagged anti-fluorescein (Fluo) single-chain Fv (ScFv) fused to the extracellular D2 domain of erythropoietin receptor (EpoR D2) on the surface of transduced cells. Added Fluo-conjugated bovine serum albumin (BSA; BSA-Fluo) was shown to act as a ligand for these CRs by inducing oligomerization, thereby enabling the activation of corresponding downstream signaling molecules [18, 20].

### Construction of a Retroviral System Capable of Highly Efficient Transduction of Murine HSCs

To obtain high-titer retrovirus supernatant, we established a series of stable virus producer cell lines based on 293GPG cells [34]. First, a retroviral packaging cell line 293GP [33] was co-transfected by lipofection with each pGCDNsam construct and with the pcDNA3.1-VSV-G encoding a VSV-G envelope gene to induce transient production of VSV-G pseudotyped retroviruses. The culture medium of the transfected 293GP cells was collected and used for transduction of 293GPG cells. Culture supernatant was collected from established transduced 293GPG cell lines as described [30] and was centrifuged at 6000 *g* for 16 h at 4 °C followed by resuspension of the viral pellet in alpha-minimal essential medium (α-MEM) to obtain virus at ~ 100-fold concentrations. Virus titers were determined by efficiency of Jurkat cell transduction.

### Transduction of Murine HSCs

**Fig. 1:**
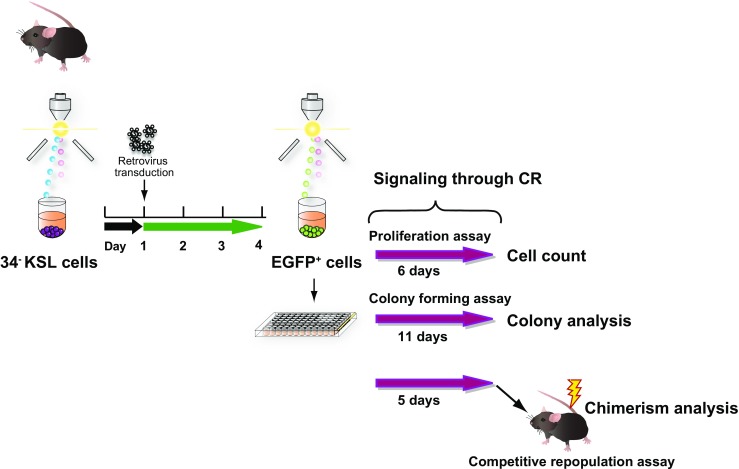
Schematic representation of the experiments. Murine CD34^−^KSL HSCs of C57BL/6 (B6)-Ly5.1 origin were sorted into 96-well plates at 600 cells/well on day 0, prestimulated with SCF + TPO, and subjected to retrovirus transduction on day 1. Three days later (day 4), the EGFP-positive cells were sorted into culture wells of 96-well plates for further assays. To test proliferative responses, cells (25 cells/well) were kept in culture for another 6 days. For colony-forming assays in liquid culture, single cells were allowed to form colonies in each well for 11 days. To test long-term reconstitution ability, cells (55 cells/well) were stimulated for 5 days in multiple wells, combined, and transplanted competitively with a fixed number of B6-Ly5.1/5.2 BM cells into lethally irradiated B6-Ly5.2 recipient mice. Of note is that test cells are expected to receive signals through CR via the artificial ligand BSA-Fluo only for the period indicated by red arrows (e.g., 5 days ex vivo for the transplantation assay)

Retroviral-mediated transduction into HSCs was carried out as reported (Fig. 1) [29]. Murine HSCs were sorted into U-bottom 96-well plates precoated with human fibronectin fragments (RetroNectin, Takara Bio, Otsu, Japan), with each well containing α-MEM supplemented with 1% FBS, 50 ng/ml mouse stem cell factor (mSCF), 100 ng/ml mouse thrombopoietin (mTPO) (Peprotech, Rocky Hill, NJ), and 50 μM 2-mercaptoethanol (2-ME; Sigma). One day later, cells were transduced with retroviral particles at a multiplicity of infection of ~ 600 for 24 h. After transduction, medium was replaced with S-clone SF-O3 (S-clone, Eidia, Tokyo, Japan) supplemented with 1% BSA, 50 ng/ml mSCF, 100 ng/ml mTPO, and 50 μM 2-ME. On day 4 of culture after transduction, cells expressing EGFP at high intensity (EGFP^+^ cells) were sorted and used for assays. In general, transduction efficiency ranged between 60% and 80% before sorting.

### In Vitro Growth Assay of HSCs

Proliferative responses were examined as reported [29]. After transduction, CD34^−^KSL HSCs were cultured for 3 days in S-clone medium supplemented with 1% BSA containing 50 ng/ml SCF and 100 ng/ml TPO (Fig. 1). EGFP^+^ cells were sorted into 96-well plates at 25 cells/well using a MoFlo cell sorter (Beckman Coulter, Indianapolis, IN). Each well contained S-clone supplemented with either 50 ng/ml SCF alone, SCF and 5 μg/ml BSA-Fluo (Sigma), or SCF and 50 ng/ml TPO. After 6 days, the cells were counted using Flow-count beads (Beckman Coulter) as described [29].

### Single-Cell Colony Assays in Liquid Culture

Our colony assay in liquid medium is described [29, 31, 35]. After being transduced as described above, EGFP^+^ cells were sorted into 96-well plates to allow clonal growth, that is, at 1 cell per well (Fig. 1). To allow colony formation, each well contained S-clone with 10% FBS and 50 μM 2-ME as basal medium. Supplemented reagents were either 10 ng/ml mSCF alone, mSCF and 5 μg/ml BSA-Fluo, or mSCF and 3 additional cytokines (10 ng/ml mTPO, 10 ng/ml mouse interleukin [mIL]-3, and 1 U/ml human erythropoietin [hEPO], all from Peprotech). After 11 days, the wells were examined and those containing over 50 cells were scored as exhibiting colony formation (max. 48 wells). Colonies also were evaluated with light microscopy for cellular composition after transfer onto glass slides using Hemacolor Rapid staining (Merck, Darmstadt, Germany) [29, 31, 35].

### Competitive Repopulation Assay

Competitive repopulation assays were performed using the Ly5 congenic mouse system (Fig. 1) [29, 31]. Based on results of the two previous in vitro assays, S-Mpl-STAT5 was selected among the 5 single-motif chimeras for an in vivo assay. The parental GCDNsam-I/E vector was used to obtain cells expressing only EGFP but no CRs (mock control, Mock). In addition, two other groups were studied: One used S-Mpl-NM as a no-motif control, the other used S-Mpl-WT as a positive control mimicking wild-type c-Mpl signaling. After transduction, EGFP^+^ cells (B6-Ly5.1) were sorted into 96-well plates at 55 cells/well and cultured for 5 days in S-clone supplemented with 50 ng/ml SCF and 5 μg/ml BSA-Fluo. Mock control cells were also cultured in the presence of SCF and 50 ng/ml TPO for 5 days as an additional control (Mock, S + T). Because the extent of cell growth varied between groups, we used the “expansion equivalent” of 458 EGFP^+^ HSCs on day 4 (the day of sorting) as transplants per recipient mouse. Each mouse thus received test cells collected from approximately 8 wells in the same condition. These cells were competitively transplanted into lethally irradiated B6-Ly5.2 mice (*n* = 7) together with 1.8 × 10^5^ competitor cells from BM of B6-Ly5.1/5.2 mice. Peripheral blood was analyzed at indicated times as described [29, 31]. For secondary transplantation, BM cells were pooled from primary recipient mice (~ 16 weeks) and transplanted into lethally irradiated B6-Ly5.2 mice (*n* = 7).

### Long-Term Donor Chimerism Analysis in Hematopoietic Cell Populations

Phenotypic cell-surface markers used to define each hematopoietic subset are summarized below. Antibodies are described in detail in Supplementary Table 1.

- Myeloid cells (peripheral blood myeloid-lineage cells): CD4^−^CD8^−^Gr1^+^Mac1^+^B220^−^
- B cells (peripheral blood B cells): CD4^−^CD8^−^Gr1^−^Mac1^−^B220^+^
- T cells (peripheral blood T cells): CD4^+^CD8^+^Gr1^−^Mac1^−^B220^−^

### Statistical Analysis

The details are described in figure legends where applicable.

## Results

Using Ba/F3 cells, we previously showed that all 7 CRs (Supplementary Fig. 1), including S-Mpl-NM, induced Jak2 phosphorylation upon BSA-Fluo-stimulation [20]. In contrast, patterns in phosphorylation of downstream molecules varied significantly for each CR, showing variable levels of specificity according to types of tyrosine motifs incorporated [20]. To apply this CR system, we transduced murine HSCs with the same retrovirus vectors, following previously reported procedures [29]. Of note is that murine HSC purity was high, matching that obtainable using SLAM family markers [29, 32]. In addition, we successfully obtained cells with ~ 100% EGFP expression by fluorescence-activated cell sorting, meaning that, the mock-vector control aside, all test HSC populations were ~ 100% positive for the expression of each CR as well. These cells were subjected to downstream assays (Fig. 1).

**Fig. 2:**
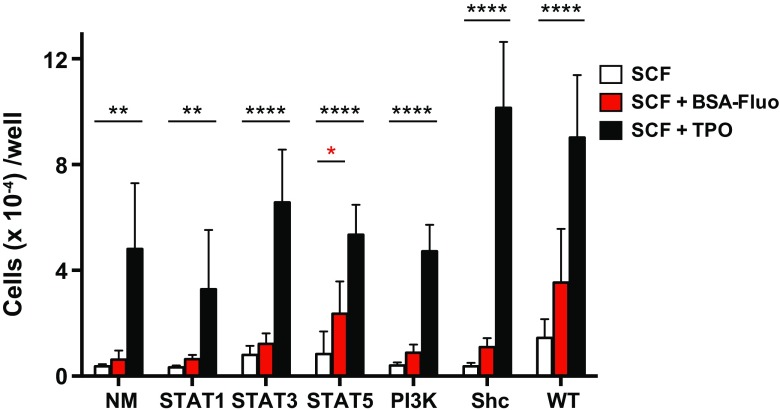
Proliferative responses in HSCs stimulated through signaling from each chimeric receptor. The cells in each well were counted 6 days after stimulation in the presence of the indicated ligands (SCF, SCF + BSA-Fluo, SCF + TPO). Shown are mean cell numbers ± SE (quintuplicate cultures) with type of CR (*n* = 5). One-way-ANOVA with Holm Sidak’s multiple comparison test was used for statistical analysis with “SCF-alone” set as control. A *p* value < *0.05* was considered statistically significant. *P < *0.05*, **P < *0.01*, ****P < *0.0001*

We first tested proliferative responses in these cells in our defined serum-free culture (Materials and methods). Consistent with previous results [11, 29], SCF alone induced minimum cell growth in all groups (Fig. 2, SCF). In contrast, SCF + TPO simulation yielded dramatic expansion for all groups with some variations in cell counts (Fig. 2, SCF + TPO). CR-mediated signaling effects, tested by the addition of BSA-Fluo together with SCF (SCF + BSA-Fluo), were observed in S-Mpl-WT receptor-expressing cells as enhanced proliferative responses greater than those achieved by SCF alone, although the difference did not reach statistical significance (*P* = *0.098*). Among the single-motif CRs, only that for S-Mpl-STAT5 exhibited significant enhancement vs. an SCF alone control (*P* = *0.046*). Considering the highly specific nature of the downstream target activation shown for this CR [20], Stat5 may in vitro have a dominant role in proliferative responses to TPO stimulation in HSCs.

**Fig. 3:**
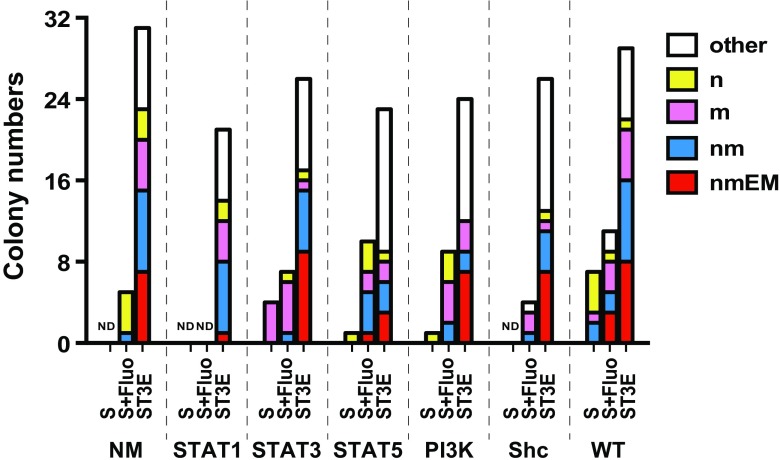
Assessment of colony formation from HSCs expressing each chimeric receptor. Shown are numbers and types of colonies formed by forty-eight single HSCs. Sorted EGFP-expressing single cells were cultured for 11 days in the presence of the indicated ligands (S: SCF alone; S + Fluo: SCF + BSA-Fluo; ST3E: SCF + TPO + IL-3 + Epo). Wells that at 11 days contained > 50 cells were scored as demonstrating colony formation. Each color represents the type of colonies classified by cellular composition. Each single letter represents a type of differentiated cells, i.e., n for neutrophils, m for macrophages, E for erythroblasts, and M for megakaryocytes. Notably, “nmEM” means formation of colonies containing all the above 4 lineage cells, showing multi-lineage potential in test cells. Other: unclassifiable colonies. ND: Colony formation not detectable. CR types are indicated below the graph

We then examined ability of signaling mediated by each CR to support colony formation from single HSCs in the presence of SCF. As shown, SCF alone induced almost no colony formation in all groups except for WT (Fig. 3, S). In contrast, the cytokine-rich condition, a positive control, induced formation of 20–30 colonies from 48 single cells irrespective of CR types (Fig. 3, ST3E). Among these colonies was at least one nmEM colony, meaning that after 4 days’ manipulation cells persisted that retained multi-lineage myeloid potential. Remarkably, BSA-Fluo stimulation was found capable of colony formation in cooperation with SCF by HSCs expressing CRs other than S-Mpl-STAT1 (Fig. 3, S + Fluo). Of note is that other than the CR for S-Mpl-WT, only stimulation with the CR for S-Mpl-STAT5 led to nmEM colony formation (Fig. 3, S + Fluo). Therefore, selectively to activate Stat5 may be preferable if HSCs are to be induced to proliferate while retaining multi-lineage progenitor abilities.

**Fig. 4:**
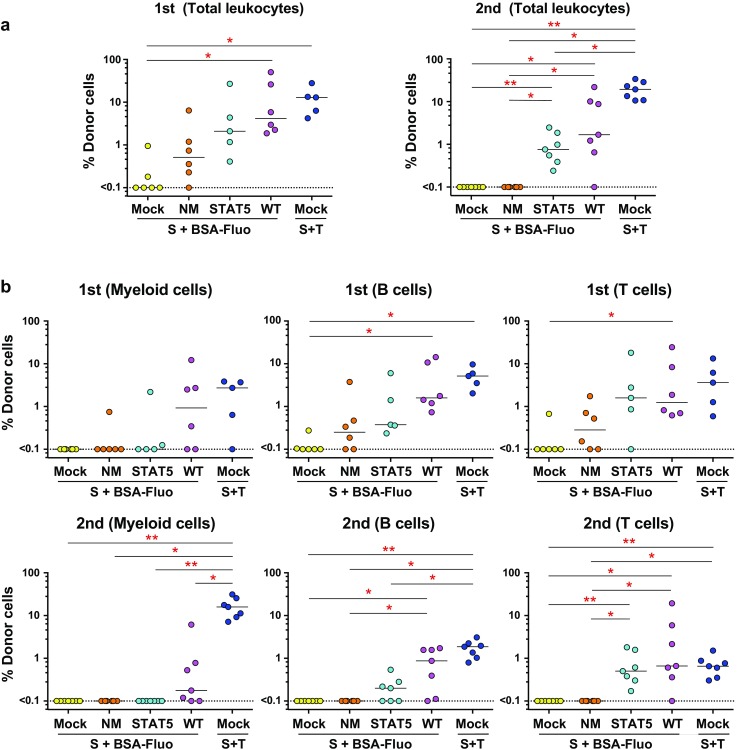
Long-term reconstituting abilities tested in HSCs stimulated ex vivo via chimeric receptors. Long-term results in competitive repopulation assays (16 weeks for primary – 1st transplantation and 17 weeks for secondary – 2nd transplantation). **a** Shown are values of % donor cell chimerism determined in total leukocytes. A minimum quantitation limit for B6-Ly5.1^+^ cell chimerism was set at 0.1%. Values below this limit were treated as 0.1 for calculation. **b** Shown are values of % donor cell chimerism determined in each indicated cell lineage. Mock represents “mock” transduction; after transduction, HSCs should express EGFP but no CR. In the other three groups, HSCs should express a type of CR (NM, STAT5, or WT) that is expected to transmit BSA-Fluo-mediated signaling into cells (S + BSA-Fluo). Another control group used mock-transduced cells cultured with SCF and TPO (Mock, S + T). Comparisons of multiple groups were performed by Steel–Dwass method. A *p* value < *0.05* was considered significant. *P < *0.05*, **P < *0.01*

We finally tested whether signaling mediated by each CR in combination with SCF could maintain in vivo reconstitution abilities in HSCs after ex vivo 5-day cultivation (Fig. 1). In this experiment, we set up 5 groups. In the first control group, HSCs during ex vivo culture received signals through endogenous c-Mpl receptors via addition of TPO (Fig. 4, Mock control cells expressing no CRs; Mock; S + T). In 3 test groups, HSCs received signals through each CR (Fig. 4, NM, STAT5, WT; S + BSA-Fluo) upon addition of artificial ligand BSA-Fluo, but not through endogenous c-Mpl. Another Mock control cohort received SCF signals alone with no other ligand-mediated signals (Fig. 4, Mock; S + BSA-Fluo). Competitive repopulation assay results showed significantly higher test cell chimerism in recipients of S-Mpl-WT-stimulated cells than in recipients of Mock SCF-alone control cells, indicating ability of this WT CR to transmit signals that support stem cell activity (Fig. 4a, 1st, *P* < *0.01*). Of note is that signals mediated by S-Mpl-STAT5 also favorably affected retention of reconstitution abilities in HSCs, whereas no-motif CR effects were only marginal (Fig. 4a, 1st, Mock vs. STAT5, Mock vs. NM). Test cell chimerism was maintained even after secondary transplantation in the recipients of S-Mpl-WT-expressing cells (Fig. 4a, 2nd, and Supplementary Figs. 2 and 3), indicating that this artificial CR could transmit signals compatible with retention of self-renewal potential in HSCs during 5-day ex vivo cultivation. Interestingly, whereas improvement over SCF-alone control (Mock) achieved by signaling via no-motif CR was completely lost in secondary recipients (Fig. 4a, 2nd, Mock vs. NM), signals from S-Mpl-STAT5 clearly rendered HSCs capable of chimerism formation in the serial transplantation setting (Fig. 4a, 2nd, and Supplementary Figs. 2 and 3, Mock vs. STAT5, NM vs. STAT5).

We then extended chimerism analysis in each lineage to know whether CR-mediated signals qualitatively affected long-term reconstitution ability in HSCs during ex vivo culture. As shown, HSCs cultured under stimulation through endogenous c-Mpl receptors with TPO in the presence of SCF showed balanced reconstitution in primary recipients (Fig. 4b, 1st, Mock, S + T) and to some extent biased reconstitution in secondary recipients, with donor chimerism higher in myeloid cells than in lymphoid cells (Fig. 4b, 2nd, Mock, S + T). HSCs cultured with S-Mpl-WT signaling also exhibited balanced reconstitution in primary recipients (Fig. 4b, 1st, WT, S + BSA-Fluo), but showed weakened, though still clearly visible, myeloid potential in secondary recipients (Fig. 4b, 2nd, WT, S + BSA-Fluo). Such lymphoid-biased reconstitution became far more evident in HSCs cultured with signals mediated by S-Mpl-STAT5, especially after secondary transplantation (Fig. 4b, 2nd, STAT5, S + BSA-Fluo). Donor-cell contribution in this group was completely lost from the myeloid-cell lineage, whereas donor-cell chimerism in T cells showed levels comparable with those established by S-Mpl-WT-expressing HSCs (STAT5 vs. WT, S + BSA-Fluo), and also with those in recipients of TPO-stimulated control HSCs (Fig. 4b, 2nd; STAT5, S + BSA-Fluo vs. Mock, S + T). These results suggest that in our experimental system dissection of signaling events affecting cell fates is feasible in primary HSCs based on the use of effector molecules positioned downstream of the c-Mpl receptor.

## Discussion

To our knowledge, this is the first report describing artificial ligand-receptor pairs capable of transmitting obvious stem cell supportive signals in culture for primary HSCs. This we believe to be an important step forward in HSC research, which requires as the gold standard assay the analysis of reconstituting ability in test cells by transplantation, generally necessitating serial transplantation to confirm test cell self-renewal ability [31]. Using this system, we showed that a complex cytokine signaling network in primary HSCs can be dissected, with singling out of the roles of individual signaling molecules. While single motif-incorporated CRs were used in this study, a strategy to construct CRs with multiple functional motifs has also been established [36]. Such CRs can activate multiple signaling molecules of interest, thereby permitting dissection of synergistic effects of signaling molecules on HSC proliferation.

As a test cell population, we throughout the study used highly-purified HSCs known to be predominantly quiescent at harvest. This was simply because we thought it appropriate to start experiments with the use of this “well-defined” cell population as a test platform for our CR systems. Recently, it has been suggested that the use of total BM cells containing cycling HSCs is highly recommended for comprehensive understanding of the biology of the entire stem cell compartment [37, 38]. It would therefore be fascinating to test our CRs for their effects on other HSC compartments within BM.

Our main focus is in modification of HSCs ex vivo to realize better transplantation outcomes. To this end, we used mock-treated HSCs engineered to express the EGFP marker alone as a positive control for a competitive transplantation assay after culture with SCF and TPO, *i.e*., stimulated through endogenous c-Mpl. These control cells showed fairly good reconstitution ability, yielding steady donor cell chimerism in secondary recipients. On closer inspection, however, the observed reconstitution pattern was myeloid-biased, consistent with that seen in aged HSCs [39]. We interpret this as evidence that our defined culture conditions [29, 31, 32] did not protect expanded HSCs from stress in culture that produced functional aging.

Preservation of lymphoid potential, but not of myeloid potential, as shown in HSCs expressing S-Mpl-STAT5 after serial transplantation, thus may have implications in transplantation medicine. For example, gene therapy for severe combined immunodeficiency diseases often requires retention of lymphoid potential at high quality in human HSCs during ex vivo manipulation [40–42]. Future experiments will find relationships between the signaling molecule preferentially activated and the reconstitution potential conferred on HSCs. Such approaches may represent an important step towards achievement of favorable ex vivo regulation of HSCs, having broad applications for various disorders that necessitate transplantation.

## Electronic Supplementary Material

Below is the link to the electronic supplementary material.

Supplementary material 1 (EPS 1995 KB)

Supplementary material 2 (EPS 6792 KB)

Supplementary material 3 (EPS 6949 KB)

Supplementary material 4 (DOCX 116 KB)
