# Supplementary material for: Dissection of Signaling Events Downstream of the c-Mpl Receptor in Murine Hematopoietic Stem Cells Via Motif-Engineered Chimeric Receptors
Source: Stem Cell Rev. 2017 Sep 25;14(1):101–9. doi: 10.1007/s12015-017-9768-7 (PMC5801400; doi:10.1007/s12015-017-9768-7)
Supplement: Supplementary file 4 — Supplementary material 4 (DOCX 116 KB) [file 12015_2017_9768_MOESM4_ESM.docx]

**Supplementary Table 1**

Antibodies used to sort donor HSCs (biotinylated antibodies)

| Antigen | Alternate name | Clone | Conjugation | Catalog # | Vendor |
| --- | --- | --- | --- | --- | --- |
| Gr-1 | Ly-6G | RB6-865 | Biotin | 13-5931-85 | eBioscience |
| Mac-1 | CD11b | M1/70 | Biotin | 13-0112-85 | eBioscience |
| Ter119 | Ly-76 | TER-119 | Biotin | 13-5921-85 | eBioscience |
| CD4 | Ly-4 | RM4-5 | Biotin | 13-0042-85 | eBioscience |
| CD8 | Ly-2 | 53-6.7 | Biotin | 100704 | eBioscience |
| CD45R (B220) | Ly-5 | RA3-7B2 | Biotin | 13-0452-85 | eBioscience |
| IL-7R | CD127 | A7R34 | Biotin | 13-1271-85 | eBioscience |

Antibodies and the reagent used to sort donor HSCs and to detect donor-derived cells in hematopoietic analysis (other conjugation)

| Antigen | Alternate name | Clone | Conjugation | Catalog # | Vendor |
| --- | --- | --- | --- | --- | --- |
| c-Kit | CD117 | 2B8 | APC | 105812B | BioLegend |
| Sca-1 | Ly-6A / E | D7 | PE | 12-5981-83 | eBioscience |
| CD34 | - | RAM34 | FITC | 110-341-85 | eBioscience |
| CD45.1 | SJL | A20 | FITC | 11-0453-85 | eBioscience |
| CD45.2 | Ly5.2 | 104 | PECy7 | 109830 | BioLegend |
| CD45R (B220) | Ly-5 | RA3-6B2 | PE | 12-0452-82 | eBioscience |
| Gr-1 | Ly-6G / Ly-6C | RB6-8C5 | APC | 108412 | BioLegend |
| Mac-1 | CD11b | M1/70 | APC | 17-0112-83 | eBioscience |
| CD4 | - | RM4-5 | PB | 100531 | BioLegend |
| CD8 | - | 5H10 | Alexa Fluor 405 | MCD 0826 | Caltag Lab. |
| Streptavidin | - |  | APC- eFluor780 | 47-4317-82 | eBioscience |

APC, Allophycocyanin; PE, Phycoerythrin; PB, Pacific Blue; FITC, Fluorescein isothiocyanate.
